# The Impact of Antibiotics and Steroids on the Nasal Microbiome in Patients with Chronic Rhinosinusitis: A Systematic Review According to PICO Criteria

**DOI:** 10.3390/jpm13111583

**Published:** 2023-11-07

**Authors:** Antonella Loperfido, Carlo Cavaliere, Elona Begvarfaj, Andrea Ciofalo, Giovanni D’Erme, Marco De Vincentiis, Antonio Greco, Stefano Millarelli, Gianluca Bellocchi, Simonetta Masieri

**Affiliations:** 1 Otolaryngology Unit, San Camillo Forlanini Hospital, 00152 Rome, Italy; 2 Department of Sense Organs, Sapienza University, 00185 Rome, Italy; 3 UOC Otorinolaringoiatria, Policlinico Umberto I, 00161 Rome, Italy; 4 Department of Oral and Maxillofacial Sciences, Sapienza University, 00185 Rome, Italy

**Keywords:** chronic rhinosinusitis, microbiome, microbiota, antibiotic therapy, steroid therapy

## Abstract

Background: The nasal microbiome represents the main environmental factor of the inflammatory process in chronic rhinosinusitis (CRS). Antibiotics and steroids constitute the mainstay of CRS therapies. However, their impact on microbial communities needs to be better understood. This systematic review summarizes the evidence about antibiotics’ and steroids’ impact on the nasal microbiota in patients with CRS. Methods: The search strategy was conducted in accordance with the PRISMA guidelines for systematic reviews. The authors searched all papers in the three major medical databases (PubMed, Scopus, and Cochrane Library) using the PICO tool (population, intervention, comparison, and outcomes). The search was carried out using a combination of the key terms “Microbiota” or “Microbiome” and “Chronic Rhinosinusitis”. Results: Overall, 402 papers were identified, and after duplicate removal (127 papers), excluding papers off-topic (154) and for other structural reasons (110), papers were assessed for eligibility; finally, only 11 papers were included and summarized in the present systematic review. Some authors used only steroids, other researchers used only antibiotics, and others used both antibiotics and steroids. With regard to the use of steroids as exclusive medical treatment, topical mometasone and budesonide were investigated. With regard to the use of antibiotics as exclusive medical treatments, clarithromycin, doxycycline, roxithromycin, and amoxicillin clavulanate were investigated. Regarding the use of both antibiotics and steroids, two associations were investigated: systemic prednisone combined with amoxicillin clavulanate and topical budesonide combined with azithromycin. Conclusions: The impact that therapies can have on the nasal microbiome of CRS patients is very varied. Further studies are needed to understand the role of the nasal microbiome, prevent CRS, and improve therapeutic tools for personalized medicine tailored to the individual patient.

## 1. Introduction

Chronic rhinosinusitis (CRS) represents a chronic inflammatory disease of the nose and the paranasal sinuses with a high prevalence in the general population (10.9% in Europe) [1]. This condition results in a significant burden on society regarding healthcare costs and lost productivity, and on the individual in terms of reduced quality of life (QoL) [2].

The nasal microbiota represents the major environmental driver of the inflammatory process in CRS, as the dysfunctional interactions that occur between microorganisms and the host immune system is known to trigger mucosal inflammation. In particular, the nasal flora dysbiosis, which means the destruction of the indigenous microbiota, can alter the integrity of the mucosal barrier, leading to the overgrowth of pathogens and inducing greater susceptibility to infections, further contributing to CRS [3,4].

In particular, in many studies on CRS, research has found a decrease in microbiome diversity and richness, as well as evenness. The reported alterations represent typical mucosal features in chronic inflammatory disorders, including CRS [5].

This deterioration may be the result of an increased presence of anaerobic bacteria that grow in biofilms [6]. Interestingly, specific works on this topic showed that in patients with CRS, the overall bacterial load was constant, while the relative richness of specific bacterial species was altered [7].

According to the literature, the microbiome in the nasal cavity of healthy adults is constituted mainly of the Corynebacteriaceae, Staphylococcaceae, and Propionibacteriaceae. However, considerable compositional variability is possible among individuals [5].

Bacterial dysbiosis represents an important biomarker of CRS. Indeed, some authors have highlighted that bacterial organisms are involved in the pathogenesis of CRS, and consequently, an alteration to the normal microbiota community of the nasal and paranasal sinus mucosa is one of the causes of CRS. Changes in the composition of microbiota can be the result of several factors, such as external and environmental triggers, which include seasonal changes, exposure to cigarette smoke, medications taken, smog, and so on; the immune status of the host; age; and intra-microbiota interactions [3,5].

Antibiotics and steroids constitute the mainstay medical treatment of CRS. Antibiotics are often prescribed to these patients to suppress pathogenic bacteria [8]. Nevertheless, it is not clear whether or not long-term antibiotic use has a positive impact on CRS patient outcomes [9]. Indeed, some studies have shown that exposure to antibiotics could be implicated in developing allergic diseases and chronic inflammation of the paranasal sinuses [10,11]. Furthermore, several authors have highlighted how prolonged exposure to antibiotic therapy can lead to an increased risk of cardiovascular events [12,13,14].

Compared to antibiotics, evidence for the efficacy of intranasal and oral steroids in the treatment of CRS with (CRSwNP) or without (CRSsNP) nasal polyps is significantly higher. There is broad consensus in the scientific community that steroids in the treatment of CRS induce significant improvements in symptoms, QoL, level of inflammatory markers, endoscopy scores, radiological scores, and reduction in nasal polyps with effects that last up to twelve weeks [15,16].

However, the impact of these therapies on nasal sinus microbial communities needs to be better understood.

Unfolding the complexity of the microbiome interactions in CRS patients will likely lead to identifying the key members of the microbial community, thus allowing researchers and clinicians to modulate the host–microbiome interaction in CRS to improve therapeutic outcomes, particularly in patients with refractory CRS (RCRS). In particular, knowing the effects of the therapies on the nasal sinus ecosystem may improve the increase in more targeted treatments for the individual patient, which is fundamental in the era of personalized medicine [17].

Therefore, this systematic review aims to summarize the evidence of the impact that antibiotics and steroids can have on the nasal microbiome in patients with CRS.

## 2. Materials and Methods

The search strategy was conducted in accordance with the Preferred Reporting Items for Systematic Reviews and Meta-analyses (PRISMA) guidelines for systematic reviews [18].

The authors searched all papers in the three major medical databases, namely, PubMed (National Institutes of Health’s National Library of Medicine—NIH NLM), Scopus (Elsevier), and Cochrane Library (Wiley), using the PICO tool (population, intervention, comparison, and outcomes) [19].

Regarding the period evaluated, all available documents on the topic from their inception until September 2023 were considered. Additionally, a manual search of the main literature on otolaryngology conferences and citation chaining was performed to avoid missing any relevant articles.

The search was carried out using a combination of the following key terms: “Microbiota” or “Microbiome” and “Chronic Rhinosinusitis”. 

The inclusion criteria for the research were represented by original article specifically focusing on the impact of antibiotics and steroid therapies on the nasal microbiome in patients with CRS, including both prospective and retrospective studies. 

Single case reports, conference papers, reviews, clinical trials, articles not in the English language, letters to the editor, articles with mixed series, and off-topic papers were excluded.

Two independent authors (A.L. and C.C.) performed a study selection, screening titles and full abstracts retrieved from each study to find eligible articles. Subsequently, the identified papers were retrieved by another author (E.B.) for full-text analysis. In cases of uncertainties about their inclusion, papers were additionally evaluated by an additional team composed of experienced specialists (A.C. and S.M.). Finally, senior experts (G.B., G.DE., M.D.V., A.G., and S.M.) provided a final evaluation and approval of the final version of the review.

The data extracted for each article were the authors, the year of publication, and the PICO items.

## 3. Results

**Figure 1:**
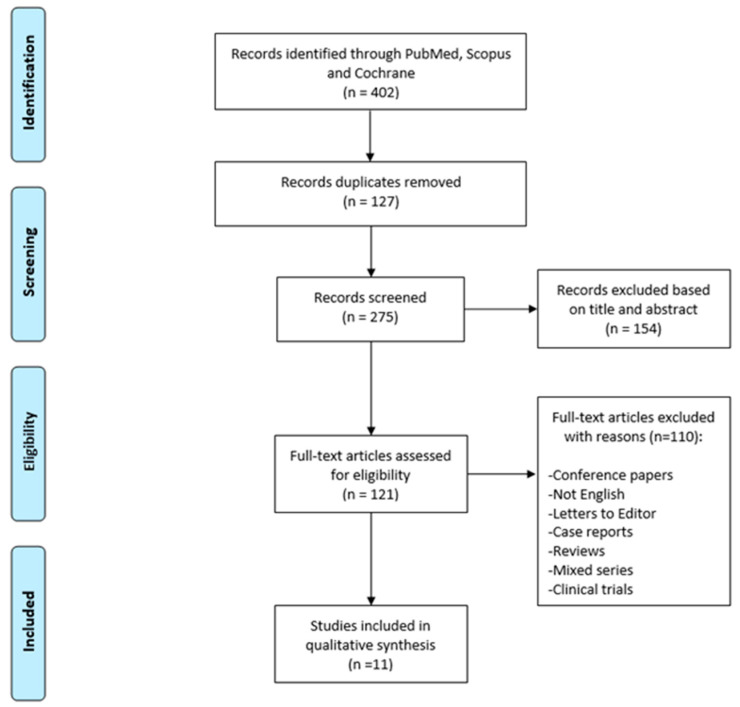
Search strategy.

The search strategy was performed according to the PRISMA guidelines as shown in Figure 1.

Overall, 402 papers were identified. After duplicate removal (127 papers), exclusion of off-topic papers (154) and for other structural reasons (110), papers were assessed for eligibility; finally, only 11 papers were included and summarized in the present systematic review. The eligible papers included in this systematic review have a publication range from 2013 to 2023. All investigated studies were published by authors from North America, Australia, New Zealand, Europe, and China.

Some authors used only steroids, other researchers used only antibiotics, and others used both antibiotics and steroids.

With regard to the use of steroids as exclusive medical treatment, topical mometasone and budesonide were investigated. Latek et al. studied the effects of intranasal corticosteroid (INC) on nasal microbiome, focusing on a study population composed of sixty-three children with CRS. The young patients were randomized to receive topical mometasone and sodium chloride (NaCl) solution (INC group) or only NaCl solution (control group) for 12 weeks. The authors found that treatment with an INC significantly increased sinonasal biodiversity. Furthermore, the authors demonstrated that the increase in bacterial diversity was correlated with the decrease in clinical symptoms, suggesting a possible causal relationship. Therefore, they conclude by stating that treatment with an INC improved the quality of life of children with CRS [20]. In another paper, Liu et al. evaluated the impact of topical budesonide and saline irrigations on the postsurgical sinonasal microbiota by examining the nasal and sinus swabs of twenty-eight controls and fourteen patients with refractory CRSwNP. They found no significant changes, concluding that nasal irrigation with saline is not associated with a specific alteration in the proportional abundance of commensal bacteria or biofilm-forming pathogens in patients with CRSwNP [21].

With regard to the use of antibiotics as exclusive medical treatments, clarithromycin, doxycycline, roxithromycin, and amoxicillin clavulanate were investigated. Chen et al. evaluated the effects of postoperative long-term low-dose oral administration of clarithromycin in patients with RCRS. Eighteen patients with RCRS were treated with low-dose (250 mg, once daily) clarithromycin for 12 weeks after Endoscopic Sinus Surgery (ESS). As a result, the authors found a decrease in *Streptococcus pneumoniae* in the microbiota, concluding that postoperative low-dose long-term oral administration of clarithromycin in patients with RCRS has a low risk of causing nasal flora imbalance and can promote mucosal epithelialization and improve clinical symptoms [22]. Siu et al. collected thirty subjects undergoing ESS for CRS and randomized them to one of three groups: doxycycline (100 mg daily for seven days); roxithromycin (300 mg daily for seven days), and control (no antibiotics). As a result of the treatments, the authors did not find any significant major bacterial community shifts or changes to bacterial load and diversity in all patient groups [23]. Lux et al. enrolled 156 CRS patients, 45 disease control patients (mostly requiring septoplasty and inferior turbinate reduction), and 35 healthy control subjects who received antibiotics (mainly amoxicillin clavulanate or doxycycline) the year before. The sinus microbiota was mainly composed of *Corynebacterium* and *Staphylococcus* in all three cohorts. Bacterial community dispersion was significantly greater in patients with CRS compared to the healthy control subjects but not disease control patients. However, the authors found that bacterial community profiles and diversity did not differ among subjects prescribed antibiotics compared to subjects who did not receive any antibiotics, regardless of disease status. As antibiotic effects have been shown to be minimal and unpredictable, the authors did not support preoperative antibiotic treatment for patients with CRS [24]. Hauser et al. collected thirteen patients with CRS undergoing ESS and treated them postoperatively with two weeks of oral antibiotics (amoxicillin clavulanate or clarithromycin if allergic to penicillin) and saline rinses. The authors examined patients’ samples from the nasopharynx, ethmoid, and anterior nasal cavities. They found that bacterial communities colonizing the ethmoid six weeks postoperatively were most similar to the anterior nostril and pretreatment sinus microbial profiles [25].

Regarding the use of both antibiotics and steroids, two associations were investigated: systemic prednisone combined with amoxicillin clavulanate and topical budesonide combined with azithromycin. Alammar et al. considered twenty-nine patients with CRSwNP and randomly allocated them to a steroids and antibiotics treatment group (sixteen patients treated with prednisone and amoxicillin clavulanate: CRSwNP-SA) or a steroid treatment group (thirteen patients treated only with prednisone: CRSwNP-S). Comparing them to fifteen healthy subjects, the authors found that, after three months of treatment, *Corynebacterium* genera increased in CRSwNP-SA while *Staphylococcus* and Gram-negative genera (*Pseudomonas*) increased in CRSwNP-S. In their conclusions, the authors stated that although both treatment options were effective in improving symptoms in the short term, they were not effective in the long term. Furthermore, they were not linked to any clear sinus microbiota response. Consequently, the authors recommend avoiding the use of antibiotics without evidence of active infection [26]. Renteria et al. evaluated changes in the RCRS patients’ nasal microbiome following a 4-month course of low-dose azithromycin, collecting forty-eight adults with RCRS. Patients were randomized to 250 mg of azithromycin or placebo three times a week for four months. During this time, daily budesonide saline irrigations were administered. The result in patients treated with antibiotics consisted of a decrease in *Staphylococcus aureus* in the nasal microbiome. Therefore, considering the pathogenic role of *Staphylococcus aureus*, the authors concluded that azithromycin in the refractory CRS population may provide an additional therapeutic solution for the control of this disease [27].

Eventually, there are three additional articles reporting about mixed medical treatments including antibiotics or steroids [28,29,30]. A full report according to the PICO criteria about all the aforementioned articles is available in Table 1.

## 4. Discussion

The human microbiome represents a heterogeneous community of microorganisms that live symbiotic relationships in human microhabitats. This entity is considered integral to maintaining the immune system and health, and due to the specificity of the microbial niche, the microbial composition varies across several anatomical locations, including the airways, gastrointestinal system, and skin [31,32].

Focusing on the airways, it has been demonstrated that the upper airway is continuously subjected to airflow from the external environment, as a healthy adult is able to breathe over 7000 L of air per day. The upper airways therefore provide critical physiological functions, such as humidifying, warming, and filtering inhaled air [33]. Since the nasal cavities communicate with the external environment through the anterior nostrils, they serve as a physical transition, providing an interface between the outside and the lower airways and gastrointestinal tracts [34].

Furthermore, along with the airflow, each individual inhales approximately 10^4^–10^6^ biological particles per cubic meter of air every day. Moreover, in addition to these bacterial cells, the upper airways are exposed to physical and chemical weathering agents, including oxygen, variable humidity, immunological, or nutritional factors. These factors are very important because they are responsible for the formation of specific microenvironments in the different districts of the upper airway, which include the anterior nostrils, the nose cavities, the sinuses, the nasopharynx, the Eustachian tubes, the middle ear cavities, the oral cavity, the oropharynx, and the larynx [35].

Consequently, all of these different microenvironments that constitute the upper airway host specific microbial communities composed of transient and resident microorganisms in varying proportions [36].

In research, the most frequent sampling sites for analyzing the microbiome of the upper airway are the anterior nostrils, middle meatus, and nasopharynx. The primary function of the nasal mucosa, which is the elimination of inhaled air, may explain the greater diversity of mucosal samples among these districts [37,38].

The surfaces of the nasal vestibule and anterior nostrils are relatively drier than the other districts of the upper airway. These parts are the most exposed to the external environment, and their epithelium includes sebaceous glands and vibrissae. These hairs capture the larger particles (>3 μm) of inhaled air, while smaller particles including microorganisms are trapped in a blanket of mucus covering the nose cavity and then transported by ciliated epithelial cells from the nose into the esophagus according to the process known as mucociliary clearance [39,40].

The middle meatus represents an area of great interest for research on the nasal microbiome, as the drainage of secretions from the anterior ethmoid, maxillary sinus, and frontal sinus converge in this anatomical district [41].

The nasopharyngeal mucosa is constituted by several crypts and folds, and its surface is characterized by pseudostratified ciliated epithelium and keratinized and nonkeratinized stratified squamous epithelia [42].

In addition, the nasopharyngeal cavity is the site of nasopharynx-associated lymphoid tissue (NALT), which consists of adenoids, the paired palatine tonsils, the paired tubal tonsils, and the lingual tonsil. These are composed of a wide variety of elements of the immune system, including macrophages, lymphocytes, and dendritic cells, and represent important sites for both detection and defense against microbes [43].

The paranasal sinuses play an important role in humidifying and warming the inhaled air. They are lined with ciliated columnar epithelium that creates mucus that drains into the nose cavities. These drainages generate local microniches characterized by specific microbial populations within the nasal fossa [44].

Interest in the olfactory microbiome is also growing [45]. In fact, recent research has shown a potential correlation between olfactory dysfunction and dysbiosis of the nasal microbiome of the olfactory area, specifically located on the roof of the nasal cavity at the lamina cribrosa [46].

If the human microbial community is imbalanced, beneficial and commensal bacteria that act against the excessive growth of pathogenic bacteria are typically lost [47].

The microbiota is influenced by several conditions, which include external and environmental factors, the host’s age and immune status, and intra-microbiota interactions. Among environmental factors, exposure to cigarette smoke, both active and passive, affects the nasal microbiome. In fact, cigarette smoke has immediate contact with the nasal mucosa resulting in direct impact on nasal flora through some mechanisms such as oxygen deprivation and antimicrobial activity. Furthermore, the toxic substances typically associated with cigarette smoke can break effective mucociliary clearance in the airways, impairing the immune responses against pathogens [3].

Compositional or functional alterations to the microbiome can occur in different anatomical districts. This dysbiosis has been linked to several chronic inflammatory disorders, such as inflammatory bowel diseases including ulcerative colitis and Crohn’s disease, and skin disorders such as atopic dermatitis, psoriasis, acne, and urticaria [48].

In addition, gut dysbiosis is known to be related to increased susceptibility to respiratory diseases and disorders of immunologic response and lung homeostasis. This pathophysiological mechanism is known in the literature as the gut–lung axis [49].

Changes in the microbiome are also highlighted in CRS, where the phenomenon explicitly affects the upper respiratory tract [50].

Bacterial dysbiosis associated with CRS is typically characterized by decreased diversity, elevated overall bacterial load, fragmentation between networks, loss of critical species, and colonization by pathobionts, such as *Staphylococcus aureus* [51,52].

It was once believed that nasal cavities were sterile in healthy people, with CRS emerging as a consequence of bacterial infection [53]. However, it is now widely known that several microbial communities colonize the healthy nasal region and act symbiotically there [54].

Specifically, the microbiome of a healthy nasal region is constituted mainly of Bacteroidetes, Firmicutes, phyla Actinobacteria and Proteobacteria with representatives of genera *Corynebacterium*, *Bifidobacterium*, *Dolosigranulum*, *Streptococcus*, *Staphylococcus*, and predominant *Moraxella* [55]. However, the majority of studies on this topic focus on the nasal bacterial component, with the possibility that other components of the nasal cavities’ microbiome, such as fungi, archaea, and viruses, are undertreated and therefore likely neglected [56].

The nasal cavities, especially the most anterior portion, are directly exposed to thousands of liters of inhaled air each day [57]. So, together with the gastrointestinal system, the nasal cavities are described as the main gateway for pollutants, inhaled pathogens, allergens, and pollen. This can cause possible imbalances in the community composition of the nasal microbial flora [58].

Research on the microbial community residing in the paranasal sinuses is increasingly growing. The capabilities of traditional culture methods have been surpassed, and thanks to advances in molecular technology, it is possible to distinguish numerous microbial species occupying host niches [59].

A work concerning the microbiome of the paranasal sinuses reported that most sinuses of patients with CRS are colonized by the bacterial families of *Pseudomonadaceae*, *Corynebacteriaceae*, *Streptococcaceae*, or *Stafilococcaceae* [60]. Further research revealed a *Corynebacterium tuberculostearicum* overgrowth and an enrichment in *Staphylococcus* in the paranasal sinuses [61]. Other authors have also isolated *Corynebacterium*, *Staphylococcus*, *Pseudomonas*, *Curtobacteria*, and *Haemophilus influenzae* as dominant bacterial species, specifically in the middle meatus of patients suffering from CRS [62,63].

CRS represents a chronic inflammatory disease of the nasal and paranasal sinuses. It affects up to 16% of the population and, although it is assumed to be an inflammatory disorder rather than an infectious one, it is important to consider bacterial contributions to the initiation and progression of inflammation [1].

Specifically, the European Position Paper on Rhinosinusitis and Nasal Polyps 2020 (EPOS 2020), provides a clinical definition of CRS in adults as a condition of inflammation of the sinuses typified by the presence for at least twelve weeks of two or more of the following symptoms: nasal discharge (anterior and/or posterior nasal drip), nasal congestion, decreased sense of smell, and facial pressure. In particular, one of the symptoms reported by the patient should be nasal congestion or nasal discharge. In addition to these symptoms, endoscopic signs of nasal polyps and/or mucus discharge and/or mucosal edema/obstruction of the middle meatus and CT scan abnormalities, such as mucosal changes within the ostiomeatal complex and/or sinuses, support this diagnosis.

With these guidelines, clinicians and researchers are experiencing a new era in the approach to this disease since, according to EPOS 2020, the classification of CRS has changed significantly. There has been a shift from a traditional phenotype classification of the disease, established by the presence (CRSwNP) or absence (CRSsNP) of nasal polyps, to an endotype classification, based on molecular biomarkers and specific pathophysiological mechanisms. Based on the underlying immunological pathophysiology, two dominant endotypes are distinguished: the type 2, related mostly to the Th2 immune response, and non-type 2 [9].

The type 2 immune pathway is defined by an overproduction of cytokines interleukin (IL)-13, IL-4, and IL-5; increased IgE; and eosinophils. Clinically, type 2 endotype is the most common in CRSwNP and is typically related to comorbid asthma, loss of smell, and reduced response to standard treatments, with a higher risk of recurrence compared to non-type 2 endotypes [64].

The non-type 2 immune pathway includes a combination of type 1 and type 3 immune reactions. In these pathways, the epithelial reaction to environmental triggers induces stimulation of dendritic cells and then differentiation of Th1 and Th17 cells, resulting in non-eosinophilic inflammation [65].

Recent studies have shown that *Staphylococcus aureus* is mainly associated with CRS and drives type 2 inflammatory responses through enterotoxin secretion or by binding to Toll-like receptor 2 (TLR2) [66,67]. Consequently, patients with CRSwNP, particularly those with comorbid asthma, are characterized by an increased relative abundance of *Staphylococcus aureus* [68]. Furthermore, *Streptococcus* and *Hemophilus* may be involved in neutrophil recruitment and IL-8 release in non-type 2 CRS [69,70].

Besides the nasal sinus microbiome disruption, there are many theories reported in the literature underlying the pathogenesis of CRS, including proinflammatory biofilms, underlying immune responses to airborne fungi, Staphylococcal enterotoxins, and host barrier disfunctions with inadequate immune responses. In particular, the final hypothesis on host barrier discontinuity is interesting because it includes all the components of all these hypotheses. Indeed, this hypothesis implies the loss of the barrier function, the colonization by bacteria and fungi, the impairment of host defense with increased local autoimmune response, and increased local innate and adaptive immune response. According to the most recent literature, treatment of CRS does not consider the underlying pathophysiology of the disease, but rather targets the downstream inflammatory response [71].

In this systematic review, the evidence on the impact that antibiotics and steroids may have on the nasal microbiome in patients with CRS is very mixed and heterogeneous.

Regarding the role of topical steroids, while Liu et al. found no significant changes in the nasal microbiome when treating patients with topical budesonide, Latek et al. demonstrated that treatment with topical mometasone had a significant effect on improving sinonasal biodiversity and improving the QoL of young patients [20,21].

Even regarding the therapeutic role of antibiotics in CRS, the conclusions of the collected papers differ. Chen et al., detecting a decrease in *Streptococcus pneumoniae*, stated that long-term oral administration at low doses of clarithromycin may have a regulatory effect on the nasal microbiota, allowing for mucosal epithelialization and improvement in clinical symptoms in patients with RCRS [22]. In contrast, Siu et al. found no significant changes in community or bacterial load, thus highlighting the poor sinonasal penetration of the drug as well as the unproven efficacy and possible impact of dysbiosis in sinuses and off-target sites. Hauser et al. also noted no significant changes, emphasizing the high degree of resilience of the microbiome. In addition, Lux et al. concluded that the unpreventable antibiotic impact on the sinus microbiota does not justify antibiotic therapy in the preoperative setting for patients with CRS [23,24,25].

Concerning the use of both antibiotics and steroids, Alammar et al. supported the avoidance of systemic antibiotics in CRS unless there is evidence of active infection, while Renteria et al. found a decrease in *Staphylococcus aureus* in the nasal microbiome in patients treated with antibiotics and concluded that azithromycin may constitute a valid therapeutic option for disease control [26,27].

Concerning studies on mixed medical treatments that include antibiotics or steroids, the authors could not ascertain whether the changes in the microbiome associated with the various treatments have clinical significance and, according to these papers, the use of systemic therapy in patients with CRS should be rationalized to minimize bacterial dysbiosis and the risk of resistance associated with antibiotics [28,29,30].

The main limitation of this review is related to the lack of uniformity in regard to the study populations. Some of the studies assessed the effects of antibiotics on the microbiome of patients affected by RCRS. RCRS is a subtype of CRS with unclear pathophysiology characterized by increased recurrence rates after sinus surgery, greater severity of symptoms, and associated comorbidities. As demonstrated by Feazel et al., sinus surgeries are associated with reduced richness of the nasal/sinus microbiome [72]. In such circumstances, it is unclear if the results reported were due specifically to the antibiotics or to changes in the nasal mucosa due to previous surgery. Furthermore, the duration of antibiotic therapy in the cited studies was very varied, ranging from 1–2 weeks in the studies of Alammar et al. [26], Siu et al. [23], Jain et al. [29], and Hauser et al. [25] to 12 weeks or more in the studies by Chen et al. [22] and Renteria et al. [27]. Another important limitation is the inability to differentiate the effects of antibiotics and/or INC from the nasal saline irrigations. The inability arises from the fact that the majority of the patients underwent nasal saline irrigation in addition to antibiotics or INC. As well as the impact of steroids and antibiotics, the improvement in ciliary function and the mechanical effect of saline irrigations on the stagnant secretions may account for the changes in the nasal microbiome.

Nevertheless, this systematic review provides a useful understanding of the effects of medical therapies on nasal sinus microbial diversity and composition, and it should support the clinician’s recommendation for appropriate antibiotics prescription to the patient with CRS.

## 5. Conclusions

The impact that therapies can have on the nasal microbiome of patients with CRS is very varied, and this may be due to the broad spectrum of microbiome patterns in CRS, the great individualization of responses to medical treatments, and thus the lack of a homogeneous posttherapy microbiota. Further studies on this topic are needed to increase knowledge on the pathophysiology of CRS, understand the role of the nasal microbiome, prevent this chronic pathology, and improve therapeutic tools for personalized medicine tailored to the individual patient.

## Figures and Tables

**Table 1:** Systematic review of selected articles according to PICO items.

Author	P Population	I Intervention	C Comparison	O Outcome
Alammar et al. 2023 [26]	Patients with CRSwNP	Prednisone + amoxicillin clavulanate or prednisone Duration: S for 3 weeks and A for 2 weeks	Healthy controls	*Corynebacterium* genera increases in CRSwNP-SA, *Staphylococcus* and Gram-negative genera (*Pseudomonas*) increase in CRSwNP-S
Latek et al. 2023 [20]	Children with CRS	Topical mometasone + NaCl solution Duration: 12 weeks	NaCl solution	INC increases nasopharyngeal microbiome richness
Chen et al. 2021 [22]	Patients with RCRS	Clarithromycin Duration: 12 weeks	Before treatment	Decrease in *Streptococcus pneumoniae*
Renteria et al. 2021 [27]	Patients with RCRS	Azithromycin + topical budesonide Durations: 16 weeks	Topical placebo + topical budesonide	Decrease in *Staphylococcus aureus*
Siu et al. 2021 [23]	Patients with CRS after surgery	Doxycycline or roxithromycin Duration: 1 week	No antibiotics treatment	No significant bacterial community shifts or changes
Cherian et al. 2020 [28]	Patients with CRS	Prednisolone or topical budesonide or doxycycline Duration: 3 weeks	Placebo	Increase of bacterial diversity in topical budesonide group
Lux et al. 2020 [24]	Patients with CRS	Amoxicillin clavulanate or doxycycline	Healthy controls	Increase of bacterial community dispersion in CRS patients
Jain et al. 2018 [29]	Patients with CRS	Doxycycline or prednisone Duration: 1 week	Patients with CRS	Variable and unpredictable changes of bacterial communities
Hauser et al. 2016 [25]	Patients with CRS after surgery	Amoxicillin clavulanate or clarithromycin and saline rinses Duration: 2 weeks	n.a.	No significant changes
Liu et al. 2015 [21]	Patients with RCRSwNP	Saline rinses ± topical budesonide	No active RS	No significant changes
Liu et al. 2013 [30]	Patients with RCRS	S ± A ± saline rinses ± INC	n.a.	Decrease in microbiota diversity and evenness

Legend. CRSwNP: chronic rhinosinusitis with nasal polyps; S: steroid; A: antibiotic; CRSwNP-SA: chronic rhinosinusitis with nasal polyps treated with steroid and antibiotic; CRSwNP-S: chronic rhinosinusitis with nasal polyps treated with steroid; NaCl: sodium chloride; INC: intranasal corticosteroid; RCRS: refractory chronic rhinosinusitis; n.a.: not available; RCRSwNP: refractory chronic rhinosinusitis with nasal polyps; RS: rhinosinusitis.
